# Multi-Institutional Patterns of Use of Tumor-Treating Fields for Patients with Malignant Pleural Mesothelioma

**DOI:** 10.3390/curroncol30060394

**Published:** 2023-05-23

**Authors:** Tugce Kutuk, Joshua M. Walker, Matthew T. Ballo, Robert B. Cameron, Jean Bustamante Alvarez, Sheema Chawla, Eric Luk, Deepti Behl, Alan Dal Pra, Neil Morganstein, Tamer Refaat, Arshin Sheybani, Christian Squillante, Jun Zhang, Rupesh Kotecha

**Affiliations:** 1 Department of Radiation Oncology, Miami Cancer Institute, Baptist Health South Florida, Miami, FL 33176, USA; 2 Department of Radiation Medicine, Oregon Health & Science University, Portland, OR 97239, USA; 3 Department of Radiation Oncology, West Cancer Center & Research Institute, Memphis, TN 38138, USA; 4 Department of Thoracic Surgery, UCLA Health, Los Angeles, CA 90095, USA; 5 Department of Thoracic Oncology, West Virginia University Healthcare, Morgantown, WV 26506, USA; 6 Department of Radiation Oncology, Rochester Regional Health, Rochester, NY 14621, USA; 7 Department of Medical Oncology, Ochsner Benson Cancer Center, Jefferson, LA 70121, USA; 8 Department of Medical Oncology, Sutter Health-Sutter Cancer Center, Sacramento, CA 95816, USA; 9 Department of Radiation Oncology, University of Miami Miller School of Medicine, Miami, FL 33125, USA; 10 Department of Medical Oncology, Atlantic Health System, Morristown, NJ 07960, USA; 11 Department of Radiation Oncology, Stritch School of Medicine, Loyola University Chicago, Loyola University Medical Center, Maywood, IL 60153, USA; 12 Department of Radiation Oncology, John Stoddard Cancer Center, Des Moines, IA 50309, USA; 13 Department of Medical Oncology, Virginia Piper Cancer Institute, Minneapolis, MN 55404, USA; 14 Division of Medical Oncology, Department of Internal Medicine, University of Kansas Medical Center, Kansas City, KS 64154, USA; 15 Department of Cancer Biology, University of Kansas Medical Center, Kansas City, KS 64154, USA

**Keywords:** malignant pleural mesothelioma, real-world experience, tumor-treating fields (TTFields), usage

## Abstract

(1) Background: The objective of this analysis was to evaluate the device usage rates and patterns of use regarding Tumor-Treating Fields (TTFields) for patients with malignant pleural mesothelioma (MPM) throughout the US. (2) Methods: We evaluated de-identified data from 33 patients with MPM enrolled in FDA-required HDE protocols at 14 institutions across the US from September 2019 to March 2022. (3) Results: The median number of total TTFields usage days was 72 (range: 6–649 days), and the total treatment duration was 160 months for all patients. A low usage rate (defined as less than 6 h per day, 25%) was observed in 34 (21.2%) months. The median TTFields usage in the first 3 months was 12 h per day (range: 1.9–21.6 h), representing 50% (range: 8–90%) of the potential daily duration. The median TTFields usage after 3 months decreased to 9.1 h per day (range: 3.1–17 h), representing 38% (range: 13–71%) of the daily duration, and was lower than usage in the first 3 months (*p* = 0.01). (4) Conclusions: This study represents the first multicenter analysis of real-world TTFields usage based on usage patterns for MPM patients in clinical practice. The real-world usage level was lower than the suggested daily usage. Further initiatives and guidelines should be developed to evaluate the impact of this finding on tumor control.

## 1. Introduction

Tumor-Treating Fields (TTFields) have emerged as a promising treatment approach for a variety of solid tumor malignancies [1,2,3,4,5]. TTFields function by delivering low-intensity alternating electric fields to tumor cells, disrupting their division and causing them to die [6]. The antimitotic effects of TTFields include the alignment of polar tubulin dimers and septin trimers with the electric field, which prevents proper mitosis. Additionally, the fields cause charged and polar molecules to accumulate at the cleavage furrow, which leads to abnormal mitosis and the formation of dysfunctional new cells. Preclinical studies have shown that human mesothelioma cells are highly responsive to TTFields at a frequency of 150 kHz, and when combined with certain chemotherapy agents, such as cisplatin and pemetrexed, a synergistic effect can be observed [7,8]. The single-arm phase 2 STELLAR study demonstrated the safety and preliminary efficacy of TTFields for the treatment of unresectable malignant pleural mesothelioma (MPM) with standard first-line pemetrexed and platinum-based chemotherapy [9]. This study reported a median overall survival (OS) of 18.2 months with modest device-related toxicity. In this prospective clinical study, a high median usage rate of TTFields in the first three months (16.3 h per day, which is 68% of the potential daily duration) was observed. Continued usage rates beyond the 3-month time point were not reported. Based on these results, TTFields therapy was made available for use under a Food-and-Drug-Administration (FDA)-approved Humanitarian Device Exemption (HDE) pathway in 2019 (H180002). The suggested device usage for patients is at least 18 h a day, which is 75% of the potential daily duration. However, in a recent real-world, single-institution study of five unresectable MPM patients treated with TTFields, median usage rates of 12.5 h per day (52%) for the first 3 months and 8.9 h per day (37%) after 3 months were reported [10]. Therefore, the objective of this analysis was to evaluate device usage rates and patterns of use throughout the United States (US) through a multi-institutional analysis and compare these with the usage rates and patterns for patients for the STELLAR trial.

## 2. Materials and Methods

**Figure 1:**
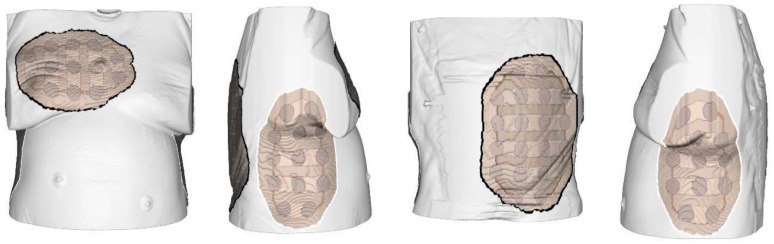
A schematic illustration of TTFields arrays applied to the thoracic region for the treatment of malignant pleural mesothelioma.

In this study, we evaluated data from 33 patients with histologically confirmed unresectable MPM enrolled in FDA-required HDE protocols at 14 institutions across the US from September 2019 to March 2022. For each patient, computed tomography (CT) scans of the chest were submitted to the device manufacturer (Novocure Ltd., Haifa, Israel) to generate a proposed layout of the arrays, which was ultimately approved by the treating physician prior to the application of TTFields according to the anatomical location of the disease and other clinical considerations (Figure 1). Patients and their caregivers were trained in the use of the device by at least one of the following: the treating physician, a designated health care provider (e.g., nurse), or a device technician (Device Support Specialist, DSS) trained by Novocure. All prescribing physicians underwent a training program and certification via Novocure. All patients were treated with a regimen of continuous TTFields at a frequency of 150 kHz [9]. The device was programmed to deliver the intended frequency in two sequential, perpendicular field directions at a maximal intensity of 1414 mA root mean square (RMS). Patients were advised to wear the device for a minimum of 18 h by the clinical team; breaks were allowed for personal needs (e.g., showering or array exchanges). Patients were instructed to replace the arrays approximately every 3 to 4 days with the help of a family member, caregiver, or DSS. During each replacement of the array, the set of arrays was moved approximately 2 cm from the previous position to ensure that the array discs were positioned in areas where there was no skin irritation. Shifting direction was determined by a trained professional (i.e., prescribing physician, designated healthcare provider, or DSS). The device logs for each patient were downloaded by the Novocure DSS every 4 weeks and compiled to assess patient usage with TTFields. No “dose” adjustments to the device were performed for adverse events. Treatment was continued until radiologic disease progression according to modified Response Evaluation Criteria in Solid Tumors (RECIST) criteria for MPM [11]; unacceptable toxicity had been reached or clinical deterioration had occurred; the treated patient requested cessation; or death.

Median and range values were used to describe continuous variables, while sample sizes and percentages were used to describe categorical variables. Comparisons of continuous variable distribution were performed using the nonparametric Mann–Whitney U test, for which values with *p* < 0.05 were considered significant. Statistical analyses were performed using SPSS version 27 (SPSS Inc., Chicago, IL, USA).

## 3. Results

Across all patients, the median number of total TTFields usage days was 72 (range: 6–649 days), and the total treatment duration was 160 months for all patients. A low usage rate (defined as less than 6 h per day, 25%) was observed in a total of 34 (21.2%) months. Four (12.1%) patients discontinued the treatment by the first month. The median TTFields device usage in the first three months was 12 h per day (range: 1.9–21.6 h), representing 50% (range: 8–90%) of the potential daily duration. Nineteen (57.5%) patients discontinued TTFields after three months. The median TTFields usage after three months decreased to 9.1 h per day (range: 3.1–17.0 h), representing 38% (range: 13–71%) of the daily duration, and was lower than usage in the first three months (*p* = 0.01). The overall usage was 10.3 h per day (range: 1.9–21.4 h), which was 43% (range: 8–89%) of the daily duration during all treatment courses. Three (9.1%) patients had a usage rate over 75% (manufacturer-suggested usage), and four (12.1%) patients had a usage rate over 68% (median usage rate in the STELLAR study). Six (18.2%) patients had a usage under the low usage rate.

**Figure 2:**
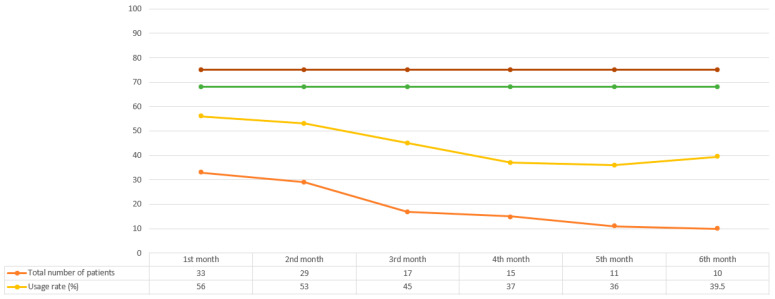
Average daily TTFields device usage percentages and the total number of patients receiving therapy by month in the first 6 months. The device usage rates recommended by the company and recorded by the STELLAR study are indicated by the brown and green lines, respectively.

Significant differences were observed in usage across months, with the highest percentage of usage observed in the first treatment month at 56% (range: 8–90%) and the lowest percentage of usage observed in the last treatment month at 32% (range: 1–74%) (*p* < 0.05). We observed discontinuation for 23 (69.7%) patients, which most commonly occurred in the first three months. The median time of TTFields treatment was 2 months (range: 1–7 months) for the physicians who treated one to four patients and 5 months (range: 1–23 months) for the physicians who treated five or more patients (*p* = 0.037). However, the median usage rate per month did not differ according to the physicians’ experience (44.5% for the physicians who treated one to four patients vs. 42% for the physicians who treated more than four patients, *p* = 0.57). The average daily TTFields device usage percentages and the total number of patients receiving therapy by month in the first 6 months are shown in Figure 2.

## 4. Discussion

This study is the first multicenter analysis of the real-world usage of TTFields—an emerging treatment approach for several solid tumor malignancies—based on usage patterns for MPM patients in clinical practice. We observed that this is a feasible treatment strategy for appropriately selected patients. The median TTFields usage rate in patients with MPM was 43%, with a low usage rate observed in a significant proportion of the treatment duration. The highest usage rate was observed in the first treatment month, with a decline in usage observed over subsequent months. Our study also highlights the importance of a physician’s experience in determining the duration of TTFields treatment, with longer treatment durations observed among physicians who treated more patients. Additionally, the real-world usage level was lower than the suggested daily usage and the results reported in the prior STELLAR study. As we know, clinical trials are generally accepted as a standard approach for generating clinical evidence for the implementation of a new treatment method. However, real-world studies can better reflect the feasibility of a treatment and the clinical scenarios affecting treatment outcomes, such as patient demographics, comorbidities, compliance, toxicities, patients’ motivation, and concurrent treatments. Additionally, as seen in this study, usage after the first three months significantly declines; therefore, the long-term usage rates for those who are responding to treatment remain a concern.

Previous studies concerning the treatment of central nervous system (CNS) malignancies indicated a significant median OS advantage with daily TTFields usage rates of ≥75% versus <75% [1,12,13]. Regarding MPM, Ceresoli et al. reported a 68% device usage rate in the first three months of the phase 2 STELLAR study [9]. This study, on the other hand, showed a median 50% device usage rate during the first three months. In addition, this study also reported device usage rates beyond the initial 3-month period, which were even lower at 9.1 h per day (38% usage rate). Similar to this study, Rivera et al. found that the median usage rates of TTFields with gemcitabine alone and TTFields plus gemcitabine and nab-paclitaxel in pancreatic cancer patients were 58% and 51%, respectively [3]. Vergote et al. also reported a median usage rate of 58% in recurrent ovarian cancer patients during the first three months [4]. Additionally, a recent case series of MPM patients treated with TTFields in combination with pemetrexed and platin-based chemotherapy also showed a median usage rate of 52% for the first three months and 37% after these three months [10]. These findings highlight the need for strategies to improve patient adherence to TTFields therapy, especially after the first three months of treatment.

Understanding device usage rates and patterns of use is critical for assessing the feasibility and effectiveness of TTFields therapy in real-world clinical settings. Our study reflects the usage rates for the real-world implementation of TTFields therapy in the treatment of MPM patients and represents an opportunity for further study and evaluation. At this point, it is crucial to identify the reasons for decreasing usage that may help further inform strategies to address issues and improve device usage rates. In a recent study, one suggested possible strategy is a fractionation approach to allow time for the sublethal repair of normal epithelial cells [10]. Another potential strategy is to customize the treatment plan and array layouts that compute the fields’ intensity with respect to the different tumor volumes to maximize treatment according to the extent of the disease, even if delivered during shorter time intervals. Finally, we found that treatment continuity was higher for the patients who received treatment from more experienced physicians and healthcare teams. Interestingly, daily usage did not differ; however, this might be related to the experience of managing side effects from the device. To overcome this issue, guidelines have recently been proposed to help improve the management of device-related skin adverse events [14,15].

One of the limitations of our study is its retrospective nature, which means there was no standardization for prior treatments, such as chemotherapy and radiotherapy regimens, across all participants. Another limitation is that the number of patients enrolled in the HDE protocols over the two-year period was limited due to the rarity of MPM diagnoses and the specific indications for TTFields treatment. In addition, given the de-deidentified nature of the initial data, clinical outcomes and patterns of failure could not be evaluated in this study.

## 5. Conclusions

In conclusion, this real-world analysis provides valuable insights into the usage patterns and feasibility of TTFields therapy for MPM patients in clinical practice. While the results indicate that TTFields therapy is a feasible treatment strategy for appropriately selected patients, the declining usage rates after the first 3 months highlight the need for further evaluation and optimization of treatment strategies. Future research should focus on identifying factors that contribute to the declining usage rates, such as patient compliance and device-related toxicities, and developing interventions to address these factors, including educational resources for physicians.

## Data Availability

Research data are stored in an institutional repository and will be shared upon request to the corresponding author.
